# Study on the correlation between the dominant microflora and the main flavor substances in the fermentation process of cigar tobacco leaves

**DOI:** 10.3389/fmicb.2023.1267447

**Published:** 2023-11-21

**Authors:** Xue Wu, Yanqi Hu, Qian Wang, Jian Liu, Song Fang, Dewen Huang, Xueli Pang, Jianmin Cao, Yumeng Gao, Yang Ning

**Affiliations:** ^1^Tobacco Research Institute, Chinese Academy of Agricultural Sciences, Qingdao, China; ^2^Graduate School of Chinese Academy of Agricultural Sciences, Beijing, China; ^3^Shandong China Tobacco Industry Limited Company, Jinan, China; ^4^Hunan Tobacco Company Chenzhou Company, Chenzhou, China

**Keywords:** cigar tobacco, macro-genome sequencing, gas chromatograph/sniffer, flavor substances, relative odor activity values, correlations

## Abstract

The flavor of cigar tobacco leaf determines the quality of finished cigar tobacco, and the enhancement of flavor generally relies on microbial fermentation. In this paper, the correlation between the dominant microorganisms and the main flavor substances of cigar tobacco leaves during fermentation and the correlation between the two were investigated to reveal the correlation between microorganisms and flavor and the metabolic pathways of microorganisms affecting the flavor substances. During the fermentation process, the main flavors of cigar tobacco leaves were sweet, light and grassy, with hexanal, 2,6-dimethylpyridine, nonanal, phenylacetaldehyde, naphthalene, and methyl benzoate as the main constituents, and the key microorganisms *Haloferax mediterranei*, *Haloterrigena limicola*, *Candidatus Thorarchaeota archaeon SMTZ-45*, the genera *Methyloversatilis*, *Sphingomonas*, *Thauera*, *Pseudomonas*, *Penicillium*, and *Aspergillus*. Correlation analysis revealed that fungi were negatively correlated with the main aroma and inhibited the main flavor substances, while bacteria were positively correlated with Benzoic acid, methyl ester in the main flavor substances, which was conducive to the accumulation of green aroma. Functional analysis revealed that the dominant bacterial population was producing aroma by metabolizing glycoside hydrolases and glycosyltransferases, performing amino acid metabolism, carbohydrate metabolism and film transport metabolism. The present study showed that the bacterial and fungal dominant microorganisms during the fermentation of cigar tobacco were influencing the production and degradation of the main flavor substances through the enzyme metabolism by the occurrence of the Merad reaction.

## 1 Introduction

In recent years, with the rapid development of China’s economy, the consumer demand for medium–and high-end cigar cigarettes is gradually increasing (Yan et al., 2021a), and the sales volume of handmade cigars in China exceeds 20 million cigars in 2021 (Yan et al., 2021b). Medium- and high-end cigar cigarettes refer to handmade cigar cigarettes, whose production process does not involve the addition of flavorings and spices and in which the volatile constituents of cigar tobacco leaves are directly originated from fermentation processes. The volatile components of cigar tobacco come directly from the fermentation process and the tobacco leaf itself (Gao et al., 2021). At present, the raw materials of cigar tobacco in China still have the problem that the main flavor is not clear and the aroma is not strong enough compared with the raw materials of Cuban cigar tobacco (Wang et al., 2020), therefore, the use of chemical means to analyze the main flavor qualitatively and quantitatively, and through microorganisms, enzymes, and some chemical reactions to enhance the flavor of cigar tobacco has become a hot spot of research (Zhao et al., 2007). It has been found that the growth and metabolism of microorganisms can cause the degradation or transformation of biomolecules such as lignin and proteins in tobacco, forming a series of volatile aroma substances, and at the same time reducing the green and heterogeneous gasses in tobacco, which will in turn enhance the quality of fermented tobacco (Su et al., 2011; Mo, 2017b). Tobacco leaves contain many microorganisms (Zhou et al., 2020) such as Bacillus, *Pseudomonas*, *Enterobacter*, *Sphingobacterium*, *Pantoea*, and *Methylobacterium* are the main genera of bacteria in tobacco leaves (Huang et al., 2010; Zhou et al., 2020). These bacteria degrade macromolecular organic matter in tobacco during fermentation. For example, *Pseudomonas* spp. can effectively degrade nicotine (Zhong et al., 2010), and *Bacillus* spp. can produce small aromatic substances by breaking down macromolecules (e.g., carotenoids; Maldonado-Robledo et al., 2003). Previous studies mainly focused on the changes of microbial community structure, the composition of volatile flavor substances and chemical substances during the fermentation of cigar tobacco leaves, and conducted some experiments on the addition of dominant microorganisms, but lacked the analysis of the main flavor and the study of the mechanism of microbial influence, which shows that it is crucial to carry out the study of the dominant microorganisms on the main flavor substances during the fermentation of cigar tobacco leaves.

The correlation between the pattern of change of dominant flora and flavor during cigar fermentation is the basis for the scientifically controllable design is cigar fermentation process. Due to the limitations of traditional isolation and culture techniques, only a small number of dominant flora can be isolated from a sample, even when a variety of media and isolation conditions are selected (Ye et al., 2021). In contrast, the combination of culture-free methods with macro-genome sequencing technology can overcome this shortcoming and detect the genomic DNA of all microscopic organisms in the samples (Shuai, 2018), which comprehensively reflects the true composition of their microbial communities. Detecting volatile metabolites in tobacco gas chromatography–mass spectrometry (GC–MS) as the most commonly used method (Cai et al., 2013; Zhang et al., 2013; Lin et al., 2014; Qin et al., 2021; Vu et al., 2021), but GC–MS/O can find the main flavor substances more accurately by taste, and the volatile compounds of lychee through the combination of GC–MS/O characterized and identified geraniol, linalool, and furanol as key aroma components of litchi (Feng et al., 2018). macro-genome sequencing technology can also systematically analyze the core microflora during fermentation and annotate the genes related to flavor substance formation. The microbiome and flavor formation-related genes of Sichuan bran vinegar were studied by macro-genome technology, and it was found that vinegar spirits possessed the basis for the formation of flavor substances through amino acid metabolism (Liu et al., 2022). Identified functional microorganisms in vinegar fermentation by correlation analysis between microbial communities and flavor metabolites (Huang et al., 2022).

In this study, we revealed the changes of microbial communities and main flavor substances during cigar fermentation by macro-genome sequencing and GC–MS/0, to understand the dominant microorganisms of cigar tobacco, and to analyze the correlation between the dominant microbial communities and the main flavor of cigar tobacco. We screened and analyzed the dominant microorganisms that have important effects on the quality and flavor of cigar tobacco leaves, with a view to producing de-stabilized microbial agents to improve the main flavor during the fermentation process of cigar tobacco leaves, and to enhance the characteristics and quality of domestic cigar tobacco leaves.

## 2 Materials and methods

### 2.1 Materials, reagents, and equipment

QX208 Cigar leaves were provided by the Tobacco Bureau of Chenzhou City, Hunan Province, and C7-C30 n-alkanes were purchased from Shanghai Sigma-Aldrich Trading Co. MS-H-Por solid-phase microextraction manual kit (Zhen Zheng Analytical Instruments Co., Ltd.), Agilent 78908B-5977A gas chromatography–mass spectrometry (Agilent, United States), 9,000 Olfactory Analyzer (Switzerland), and 57,348-U BVB/CAR/PDMS solid-phase microextraction fiber tip (50/30 micron), SUPERO, United States were used. DyNA Quant 200 Concentrometer, United States, Pharmacia Biotech Agarose Gel Electrophoresis, United States, Bio-Rad QuantiFluorTM-ST Blue Fluorescence Quantification System, United States, Promega GDS Gel Imaging System, United Kingdom METTLER TOLEDO Ice Maker, Japan, Sanyo PCR Instrument, United States, ABI.

### 2.2 Experimental methods

#### 2.2.1 Experimental pre-treatment

Constant humidity and initial temperature are set at 70% and 32°C, respectively. In addition, after fermentation at constant temperature for 10 days, the temperature was raised by 2°C for a total of 80 days, and every 10 days was a fermentation stage from FA-FI to 9 fermentation stages. At the same time of each heating sampling, the four vertices and the center of the bag from the top to the bottom of the 300 g cigar leaves, fully mixed. Approximately 50 g of samples were placed in 50 mL sterile centrifuge tubes and vortex oscillated after addition of PBS buffer (PBS concentration and PH requirements: 1x PBS, PH 7.4); Microorganisms were allowed to fully shed from the surface of the object and accumulate in PBS buffer, the buffer was stored in a sterile centrifuge tube after filtration with a filter membrane and then stored at −80° C, and the dry ice transport sample was sent to Allwegene Tech. The company carries out the extraction and detection of microbiome DNA. Dry and crush 100 g of the sample and sift through 60 mesh. Add smoke (0.5 g; to 0.001 g), place 105 ppm phenylethyl acetate (internal standard) in a 25 mL headspace bottle, seal and set aside, and repeat each set three times.

#### 2.2.2 HS-SPME-GC–MS/O analytical condition

HS-SPME analysis conditions: The temperature and heating equilibrium time of the solid-phase microextraction (SPME) manual device were 70°C and 30 min, respectively; in addition, the extraction was carried out with a 50/30 μ m DVB/CAR/PDMS extraction head for 30 min.

GC–MS conditions: A DB-5MS quartz capillary column (30 m × 0.25 mm, 0.25 μ m) was used. The high purity helium was used as the carrier gas, and the flow rate of the column was 1.6 mL/min (constant flow mode); the temperature of the injection port was 240°C; the non-split injection mode was used, and the injection volume was 2 μ L. The initial temperature was 40°C, and the temperature was kept at 40°C for 2 min, and then it was increased to 220°C at a rate of 6°C/min. Finally, the temperature was increased to 280°C at 20°C/min for 10 min. The ion source used was an ionization source with an ionization voltage of 70 eV and an ion source temperature of 230°C, respectively. The transmission line temperature was 290°C. The scanning mode was full scan mode with a scanning range of 33–325 amu. Qualitative analysis was performed based on the total ion flow diagram, peak times, spectral libraries (NIST17 library), and retention indices. Phenethyl acetate internal standard was used for quantitative analysis.

The mass spectrometry quadrupole temperature was 150°C. The electron bombardment ion source, transmission line temperature (290°C), electron energy (70 eV) and scanning range were the same as the MS conditions. The shunt ratio of the olfactory port to the mass spectrometry terminal was 1:1, and the temperature of the olfactory port was 280°C. To avoid subjectivity and to record olfactory sensation characteristics and retention times, the GC–MS/O analysis was performed by five members of the same sample for olfactory description.

#### 2.2.3 Macro-genome sequencing DNA extraction methods for cigar tobacco leaves

Extraction of microbial DNA reference E. Noah. N. A. Soil DNA Kit (Omega Bio-tek, Inc.,) [Omega E. Noah. N. A. Stool DNA Kit (Omega Bio-tek, Inc.,)]. The size and quality of extracted DNA were analyzed by electrophoresis on 1% agarose gel. The cigars that met the sequencing requirements were stored in a refrigerator at −80° C, and the unqualified samples were re-extracted. Hiseq libraries were constructed, library fragments were screened using 2% agarose gel, blocks of 400–450 BP fragments were excised for purification, then PCR enrichment was performed, and finally machine sequencing was performed on the Illumina Hiseq platform.

### 2.3 Data handling

#### 2.3.1 Relative odor activity value

The relative odor activity value (ROAV) was used to assess the contribution of each volatile component to the flavor of cigar tobacco samples, following the calculation method of Liu et al. (2008), where ROAV>1 indicates that the component contributes the most to the flavor of the sample and is the key flavor component; 0.1 ≤ ROAV<1 indicates that the component can change the flavor of the sample; and ROAV<0.1 indicates that it has no significant effect on the flavor of the sample. ROAV<0.1 means that this component has no significant effect on the flavor of the sample. Within a certain range, the larger the ROAV, the greater the contribution of the substance to the overall flavor.

#### 2.3.2 Analysis of macro-genomic data

Firstly, the original data were segmented, and the valid data were obtained by quality cutting. The reads of all samples were merged and assembled using the mosaic software MEGAHIT (or IDBA) based on the De-Brujin graph principle (Li et al., 2015, 2016), the De-Brujin graph was constructed according to the overlap relationship between the KMERS, and the Contigs above 800 BP were screened for statistical analysis and used for follow-up analysis. The ORF prediction of the stitched Contigs sequence was performed using Prodigal (Hyatt et al., 2010) software, the sequences were translated into amino acid sequences, and the non-redundant gene cataloge was obtained by CD-HIT software. The clean reads of each sample were compared with the non-redundant gene set (95% identity) by Bowtie2 software, statistical information on the abundance of genes in corresponding samples (Qin et al., 2010; Cotillard et al., 2013; Le et al., 2013; Villar et al., 2015; Zeller et al., 2015). The non-redundant genes were compared with KEGG, eggNOG and CAZy functional databases by Diamond Software, and the annotation of E < 1e-5 was selected to screen the proteins with the highest sequence similarity, so as to obtain the functional annotation information for each sequence alignment result, the alignment result with the highest score (one HSP > 60 bits) was selected for follow-up analysis (Bäckhed et al., 2015).

The data were processed by SPSS 27 software, the microbial data were analyzed by Allwegene Cloud Platform, and the Origin 2019 software was used to draw the grid map and correlation heatmap. The experimental results are presented as mean ± error.

## 3 Results

### 3.1 Dominant flavor analysis of cigar tobacco during the fermentation process

**Figure 1:**
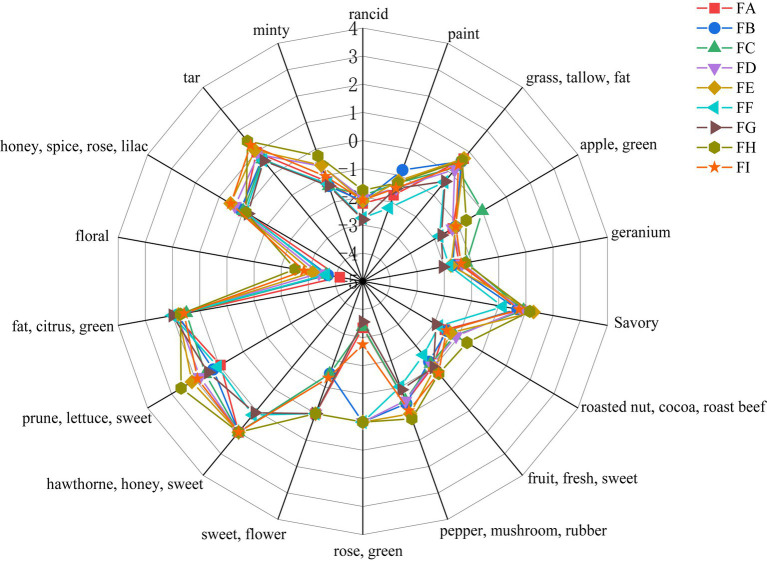
Analysis of smell results.

The aroma of cigar tobacco is produced by a combination of volatile substances, and those that can be detected by the human olfactory organs are unique to cigar tobacco. According to the GC-O olfactory intensity of the aromas and analyzed using the Origin heat map (Figure 1), the main aromas of cigar tobacco at all stages of fermentation are honey-like sweetness, cocoa sweetness, citrus-minty freshness, and grassy herbaceousness, supplemented by wood, spice (slightly spicy and peppery), floral, cocoa, and fruity aromas.

The level of odor intensity of olfactory perception does not indicate the degree of its contribution to the overall flavor of the sample, but also needs to be evaluated in conjunction with the threshold value of the flavor substances, which is different for different compounds. Some flavor substances, although their relative content is very low, contribute a lot to the overall flavor due to their low threshold value, and the compounds with a lower threshold value are more likely to be perceived by the olfactory organ when their relative content is certain. The odor thresholds of 18 volatile substances were found by GC–MS analysis of olfactory-perceived substances and by reviewing the aroma thresholds of volatile flavor substances that have been reported in books and related literature (Van Gemert, 2011; Table 1).

**Table 1:** Aroma thresholds of volatile flavor substances in cigarillos during fermentation.

Retention time	Aroma	Substance	CAS	Threshold(medium-air; mg/Kg) Van Gemert (2011)	ROAV (mg/Kg)								
					FA	FB	FC	FD	FE	FF	FG	FH	FI
9.22	Rancid	Pyridine	110–86-1	2	0.006	0.007	0.011	0.009	0.007	0.002	0.002	0.017	0.008
10.06	Paint	Toluene	108–88-3	0.527	0.018	0.162	0.052	0.034	0.063	0.006	0.033	0.053	0.033
10.53	Grass, tallow, Fat	Hexanal	66–25-1	0.005	4.865	3.645	1.989	1.548	5.013	0.401	0.431	4.064	2.454
12	Apple, green	2-Hexenal, (E)-	6,728-26-3	0.11	0.057	0.076	0.000	0.056	0.078	0.016	0.020	0.214	0.077
12.63	Geranium	o-Xylene	95–47-6	0.450	0.035	0.020	0.065	0.045	0.017	0.015	0.009	0.058	0.041
12.88	Savory	Pyridine, 2,6-dimethyl-	108–48-5	0.003	6.477	4.828	8.274	6.626	19.23	1.421	0.000	13.874	5.612
13.66	Roasted nut, cocoa, roast beef	DIMETHYL PYRAZINE	108–50-9	0.718	0.027	0.033	0.071	0.079	0.047	0.016	0.011	0.230	0.032
13.92	Fruit, fresh, sweet	hexanoic acid methyl ester	106–70-7	0.07	0.091	0.054	0.067	0.080	0.112	0.026	0.097	0.193	0.185
15.54	Pepper, mushroom, rubber	5-Hepten-2-one, 6-methyl-	110–93-0	0.068	0.362	0.412	0.324	0.325	1.029	0.096	0.125	1.582	0.775
16.71	Rose, green	1-Hexanol, 2-ethyl-	104–76-7	25.482	0.000	0.000	0.000	0.000	0.000	0.000	0.000	0.000	0.002
17.07	Sweet, flower	Benzyl alcohol	100–51-6	2.5462	0.000	0.031	0.032	0.000	0.000	0.000	0.000	0.000	0.045
17.39	Hawthorne, honey, sweet	Benzeneacetaldehyde	122–78-1	0.0063	100	100	100	100	100	15.75	12.56	100	100
18.74	Prune, lettuce, herb, sweet	Benzoic acid, methyl ester	93–58-3	0.001	8.952	18.28	38.748	63.78	140.9	11.64	29.78	395.63	83.180
18.79	Fat, citrus, green	Nonanal	124–19-6	0.0011	80.397	65.59	30.849	69.68	60.54	100.0	100.0	50.910	37.333
19.91	Floral	Isophorone	1,125-21-9	200	0.000	0.000	0.000	0.000	0.001	0.000	0.000	0.003	0.001
20.07	Honey, spice, rose, lilac	Phenylethyl Alcohol	60–12-8	0.5642	0.000	1.298	0.000	2.269	3.435	0.819	0.646	0.768	3.321
21.16	Tar	Naphthalene	91–20-3	0.006	9.696	4.772	4.526	7.666	12.01	4.106	3.867	31.751	21.024
34.52	Minty	Hexadecanoic acid, methyl ester	112–39-0	>2	0.059	0.040	0.040	0.224	0.246	0.047	0.041	0.550	0.095

In this study, only the flavor substances for which thresholds could be found were analyzed. Phenylacetaldehyde was the flavor substance with the highest relative amount and low threshold value in the FA-E and FH-I fermentation stages, and nonanal was the flavor substance with the highest relative amount and low threshold value in the FF-FG fermentation stage; therefore, these two substances were defined as the standard flavor substances (Tstan = 100).

The ROAV value is an index that can quantify the contribution of volatile flavor components to the overall aroma of the sample, and the ROAV value was calculated according to 2.2, and the results are shown in Table 1. There were six flavor compounds with ROAV>1. Hexanal, 2,6-dimethylpyridine, nonanal, phenylglyoxal, naphthalene, and methyl benzoate were the main flavor compounds of the cigar tobacco, and the six aroma compounds were classified into floral, green flavor, cheesy flavor, and green flavor according to the aroma characteristics. The six aroma compounds are classified into five categories according to their aroma characteristics: floral, green flavor, cheesy, sweet, citrus, and caramel-nutty.

### 3.2 Study on dominant microflora during the fermentation of cigar tobacco leaves

#### 3.2.1 Analysis of the dominant flora of the archaeal community in the fermentation process

**Figure 2:**
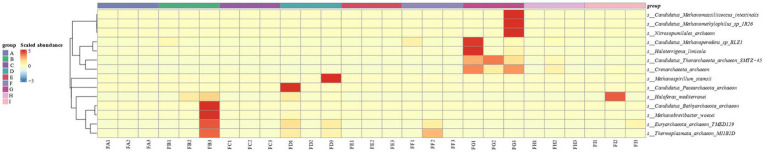
Heat map of archaebacterial species abundance clustering at the species level in different fermentation stages of cigar tobacco.

Based on the abundance data of the species level of the Archaebacteria (biology) in cigar tobacco leaves at different fermentation stages were analyzed using heatmaps in the R language, as shown in Figure 2. There was no archaeal community on the surface of cigar tobacco leaves before fermentation, and the archaeal community appeared gradually after fermentation. The changes in the abundance of archaea were relatively large in the FB, FD, FG, and FI stages of fermentation, and the changes in the strains of archaea were mainly concentrated on the *Candidatus* species.

**Figure 3:**
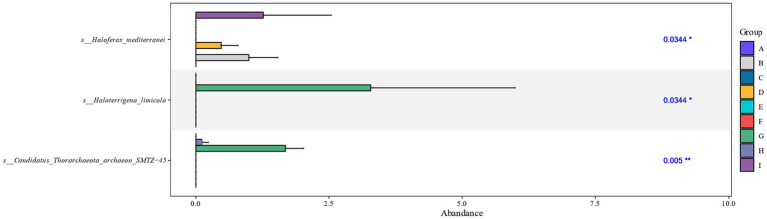
Kruskal-Wallis analysis of cigar tobacco at different fermentation stages based on species level. The horizontal coordinates of the bar graph indicate the relative abundance of a species in different subgroups, the vertical coordinates indicate the difference species, and different colors indicate different groups.

Differential analysis of the archaeal communities present on the surface of cigar tobacco leaves at different fermentation stages showed that the fermentation stages in which there were significant differences between species after cigar tobacco fermentation were mainly the FB, FD, FG, FH, and FI fermentation stages, with significant differences in the *Haloferax_mediterranei*, *Haloterrigena_limicola*, and *Candidatus_Thorarchaeota_archaeon_SMTZ-45* colonies (Figure 3).

**Figure 4:**
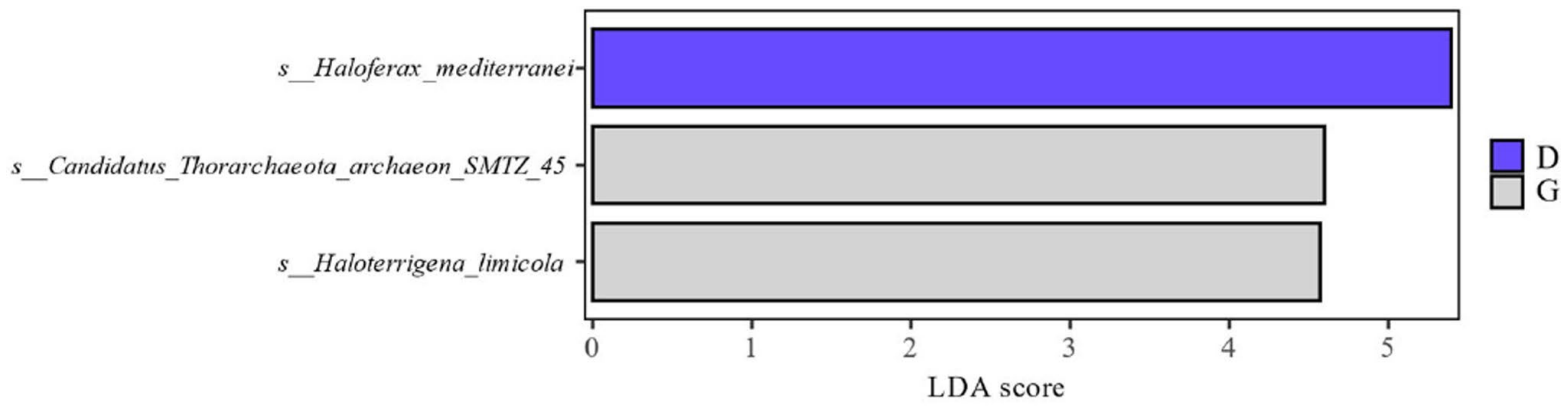
Distribution of LDA values of archaea at different levels during different fermentation stages of cigar tobacco leaves.

Combined with the relative abundance and LDA score ≥ 2.5 at the species level in Figure 4, it can be seen that during the late fermentation *Haloferax-mediterranei*, *Haloterrigena-limicola*, *Candidatus-Thorarchaeota-archaeon-SMTZ-45* the most abundant archaeon in the colony and the key archaeon colony in the fermentation of cigar tobacco leaves.

#### 3.2.2 Analysis of the dominant flora of the bacterial community in the fermentation process

**Figure 5:**
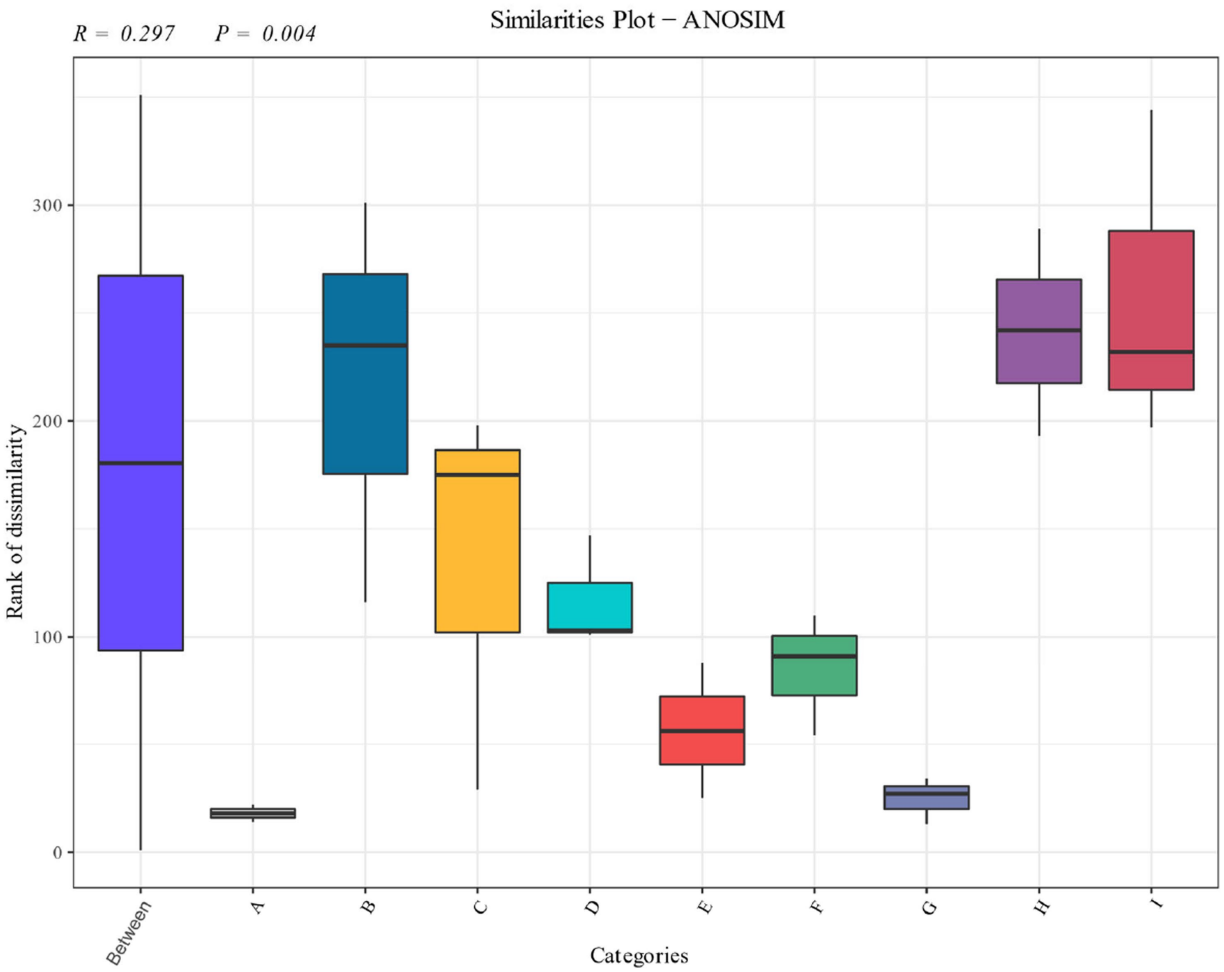
Anosim analysis of cigarillos at different fermentation stages based on species level. *R*-value is between (−1, 1), R-value is greater than 0, indicating significant difference between groups, and the confidence of statistical analysis is expressed by *p*-value, *p* < 0.05 indicates statistical significance.

Significance analysis of the bacterial community of cigar tobacco leaves in different fermentation stages was carried out as shown in Figure 5. The distribution shows that the bacterial community of the pre-fermentation samples differed significantly from that of the post-fermentation samples, and each fermentation stage occupied a different quadrant. The results showed that there were significant differences in the structure of the bacterial communities at different stages.

**Figure 6:**
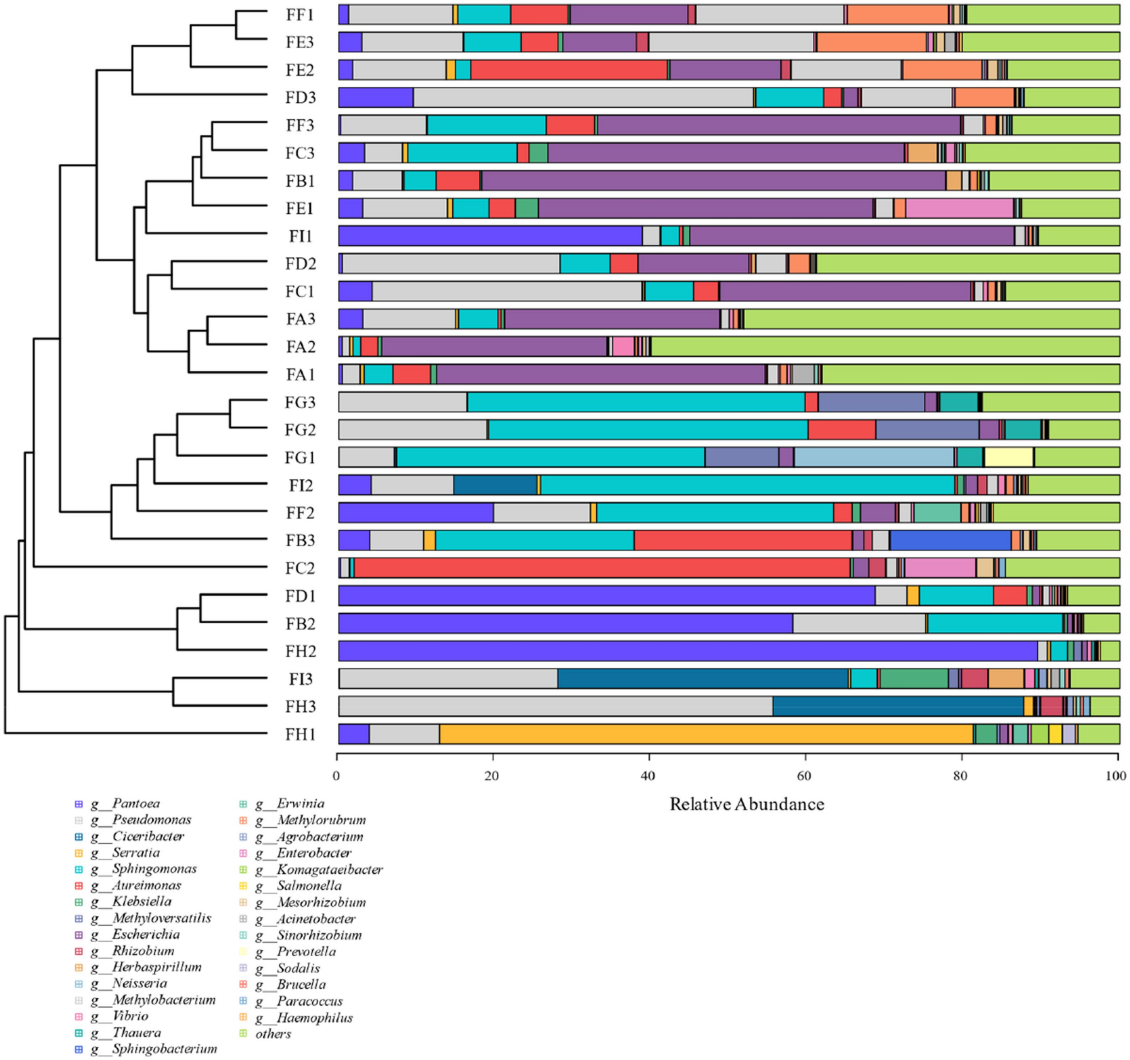
Cluster analysis of horizontal bacterial species of cigar tobacco genus at different fermentation stages.

**Figure 7:**
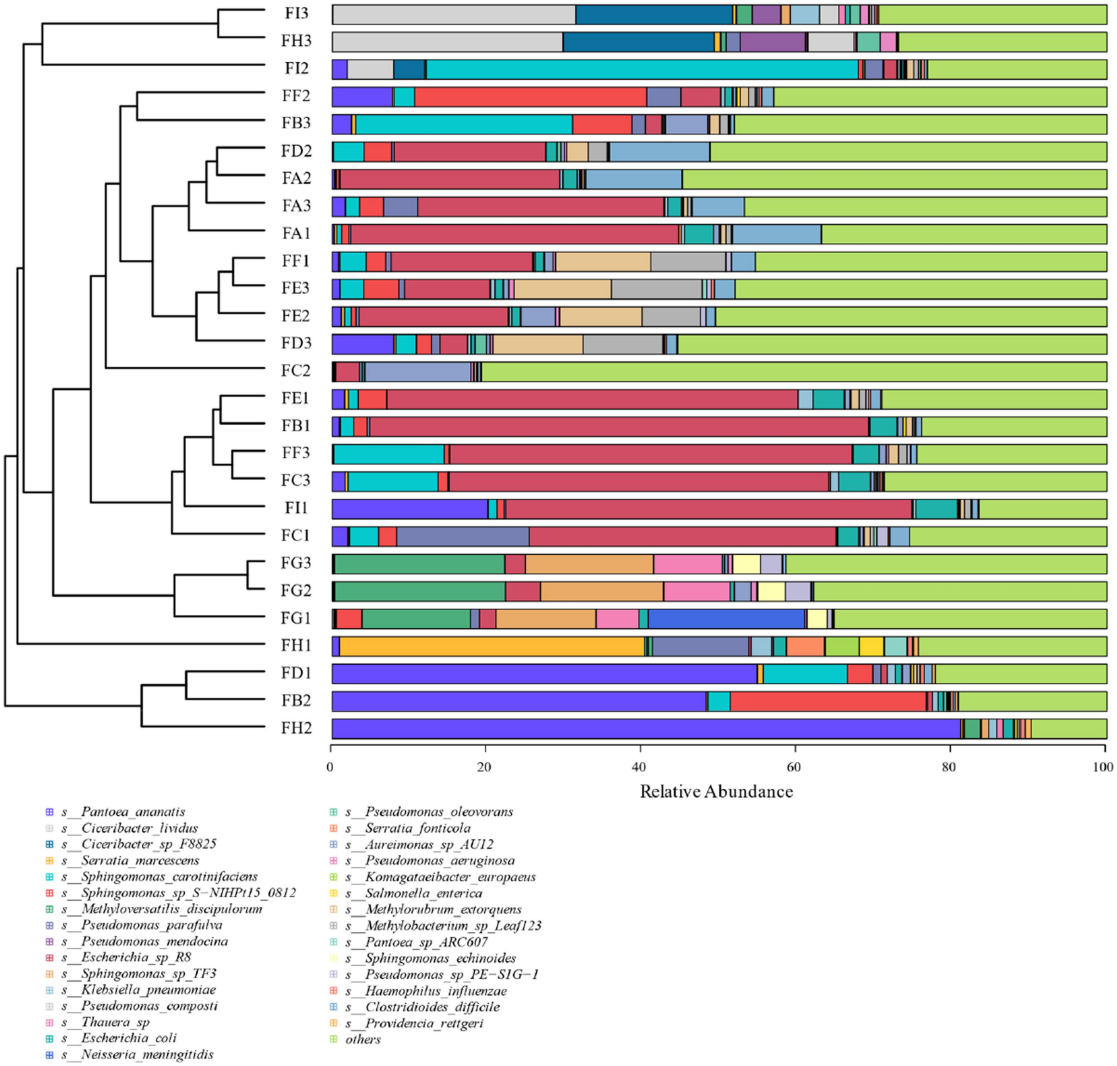
Cluster analysis of bacterial species at the level of cigar tobacco species at different fermentation stages.

Based on the 97% similarity, all sample sequences were clustered, and based on the relative abundance information of all samples at the genus level and species level (the top 30 overall abundance species were taken here), the relative abundance of species at different levels was analyzed for variation, and the abundance share of bacterial communities was obviously different among the fermentation stages; the analysis of the results at the genus level in Figure 6 revealed that Serratia, *Methyloversatilis*, *Neisseria*, *Thauera*, *Sphingobacterium*, *Prevotella*, *Sodalis*, and *Pseudomonas* had high coefficients of variation, with *Methyloversatilis*, *Neisseria*, *Thauera*, *Prevotella* genus group of species showed a gradual increase in microorganisms with the extension of fermentation time. From the analysis of the results of the seven species levels in Figure 7, it was found that *Serratia_marcescens*, *Methyloversatilis_discipulorum*, *Sphingomonas_sp_TF3*, *Thauera_sp., Neisseria_meningitidis*, *Serratia_ fonticola*, *Komagataeibacter_europaeus*, *Sphingomonas_echinoides*, and *s__Pseudomonas_aeruginosa* had high coefficients of variation, and all bacteria with high coefficients of variation were present in the high coefficients of variation except for *Komagataeibacter_europaeus*. *Komagataeibacter* europaeus is a gram-negative bacterium belonging to the genus *Komagataeibacter* of the family *Acetobacteraceae*, a common acetic acid bacterium capable of converting alcohols into acetic acid. *Komagataeibacter* europaeus is also capable of producing other beneficial metabolites such as polysaccharides and cellulases, which have potential applications.

**Figure 8:**
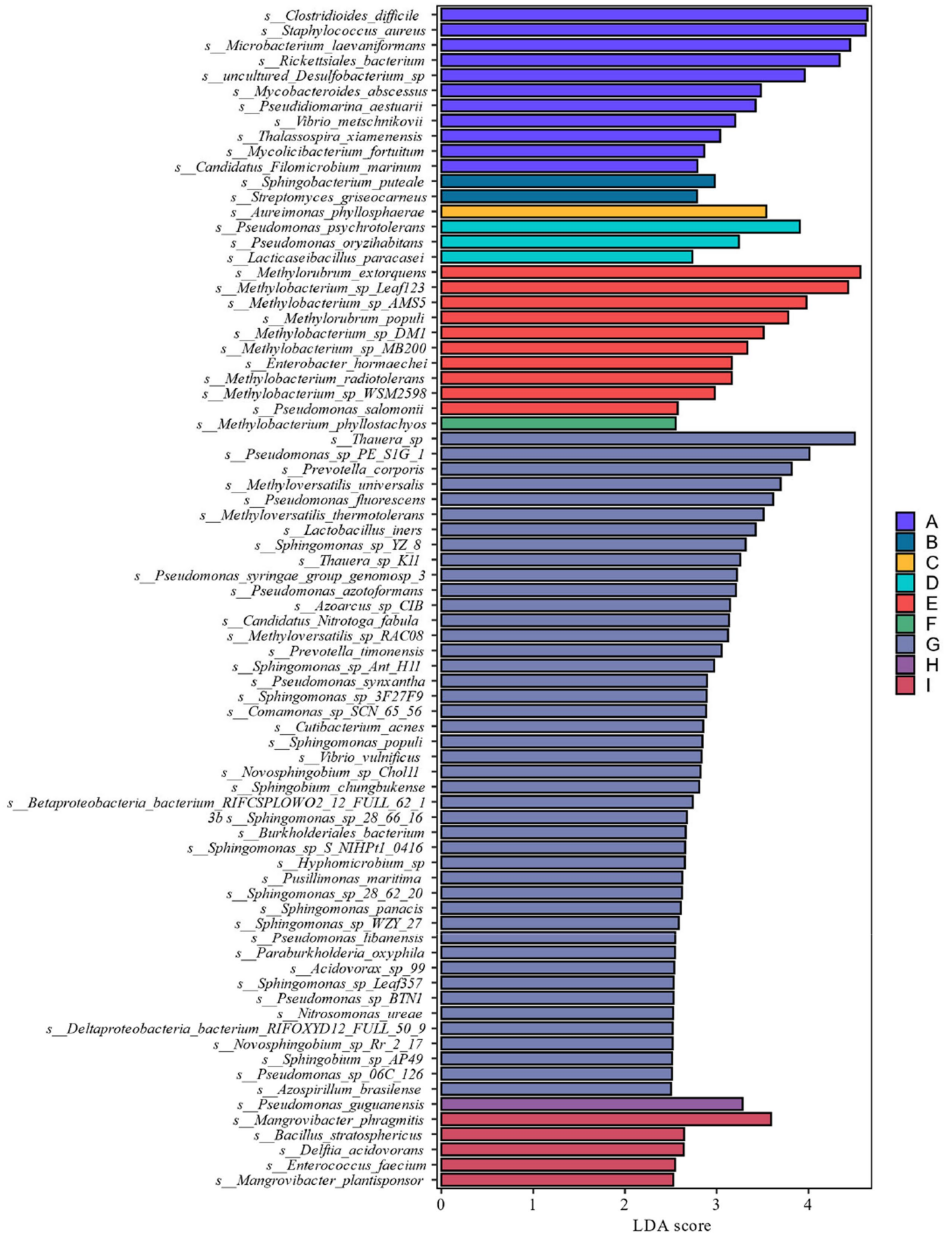
LDA value distribution of different species at different levels during different fermentation stages of cigar tobacco leaves.

From the analysis of LDA score ≥ 2.5 of the bacterial community at the species level of Figure 8, it can be seen that the genera *Pseudomonas*, *Methylorubrum*, *Sphingomonas*, *carotinifaciens*, and *Thauera* were the most abundant bacteria in the late fermentation stage of FG and FH fermentation. Combining the results of the relative abundance analyzes in Figures 6, 7 with the LDA score ≥ 2.5 at the species level in Figure 8, it can be seen that the genera *Methyloversatilis*, *Sphingomonas*, *Thauera* and *Pseudomonas* are the key bacterial colonies during the fermentation of cigar tobacco leaves.

#### 3.2.3 Analysis of dominant flora of fungal communities during fermentation process

**Figure 9:**
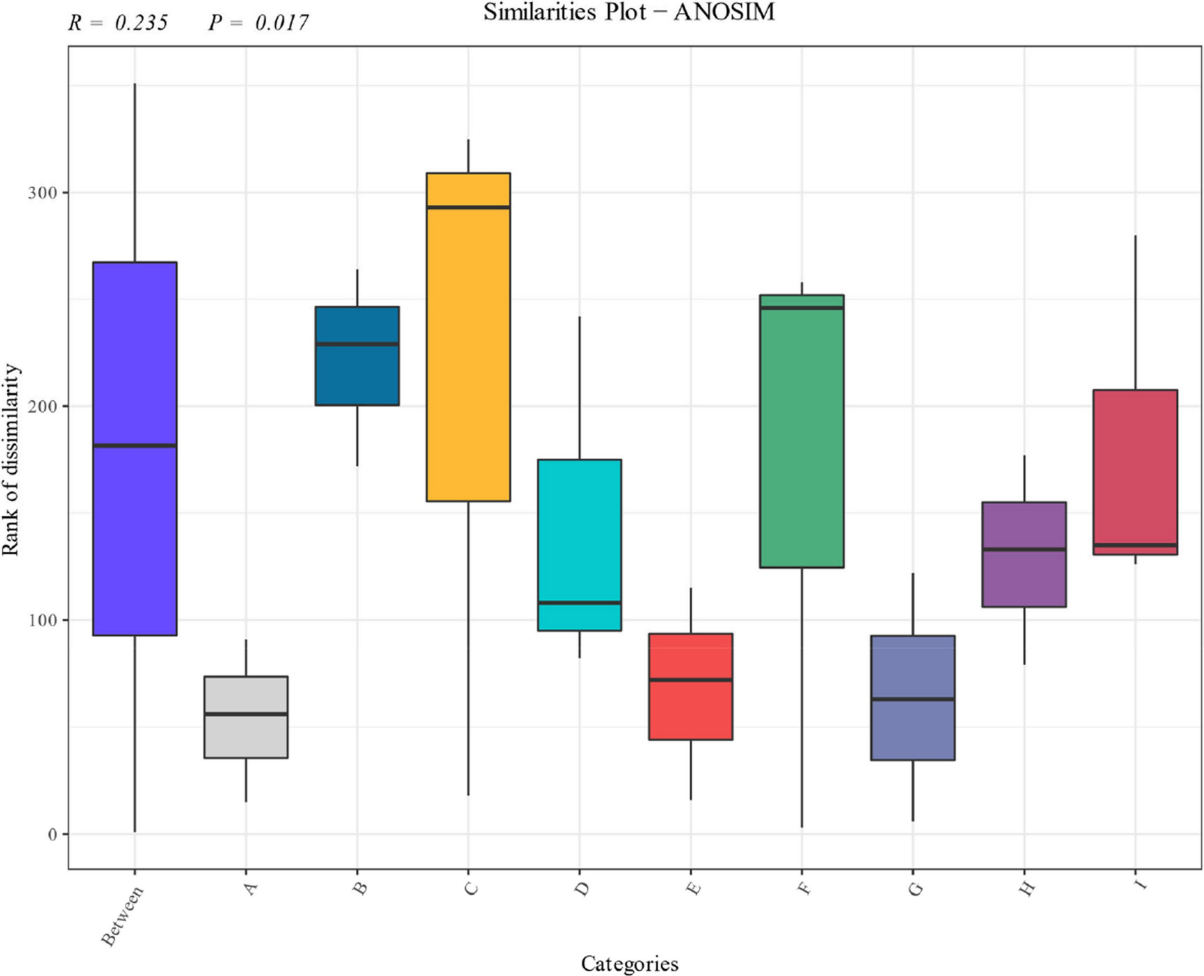
Anosim analysis of cigarillos at different fermentation stages based on species level. *R*-value is between (−1, 1), R-value is greater than 0, indicating significant difference between groups, and the confidence of statistical analysis is expressed by *p*-value, *p* < 0.05 indicates statistical significance.

Anosim analysis of the fungal community at the species level during the fermentation of cigar tobacco is shown in Figure 9, which shows significant differences in the composition of the fungal community at the species level during the fermentation of cigar tobacco. As can be seen from the distributions, there were significant differences between the fungal communities of the samples before fermentation and the samples after fermentation, and the fungal communities in tobacco leaves at different stages of fermentation occupied different quadrants. The results showed that there were significant differences in the structure of fungal communities at different stages.

**Figure 10:**
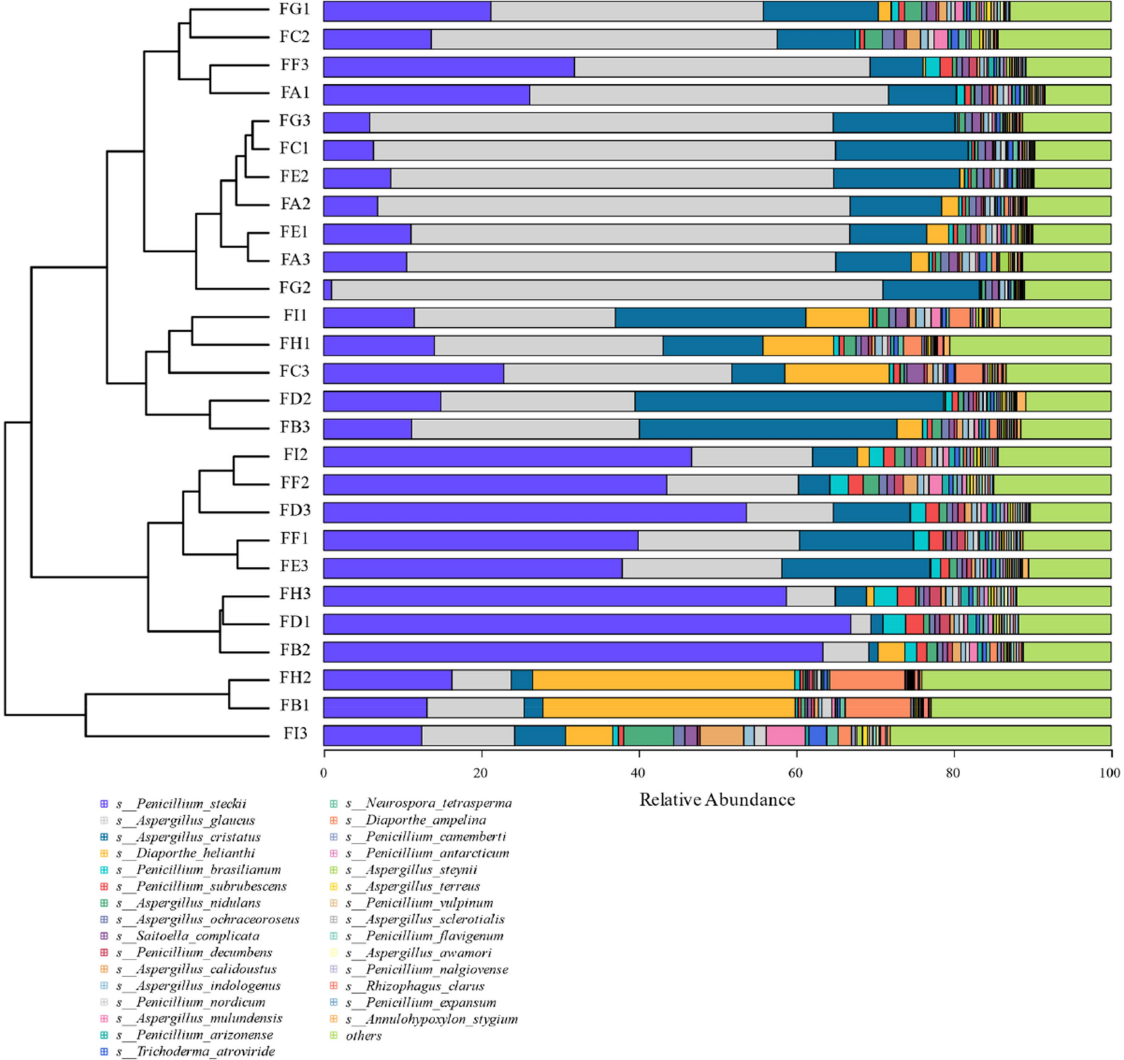
Cigar tobacco species level fungal species clustering analysis at different fermentation stages.

Based on the relative abundance of fungi at the species level (the top 30 species in terms of overall abundance were taken here) and the degree of variation of all the samples, it can be seen that the variation of the fungi *Diaporthe_helianthi* and *Diaporthe_ampelina* was more obvious (coefficient of variation >1) during the fermentation process, and the most abundant fungi were *Penicillium_steckii* and *Aspergillus_glaucus*, followed by *Aspergillus_cristatus* and *Diaporthe_helianthi*. *Penicillium_steckii* and *Aspergillus_glaucus*, followed by *Aspergillus_cristatus* and *Diaporthe_helianthi*, and the highest abundance was found in *Diaporthe_ampelina*, *Aspergillus_ampelina* and *Penicillium_glaucus* during fermentation. Genus types of fungi had higher relative abundance during the fermentation process (Figure 10).

**Figure 11:**
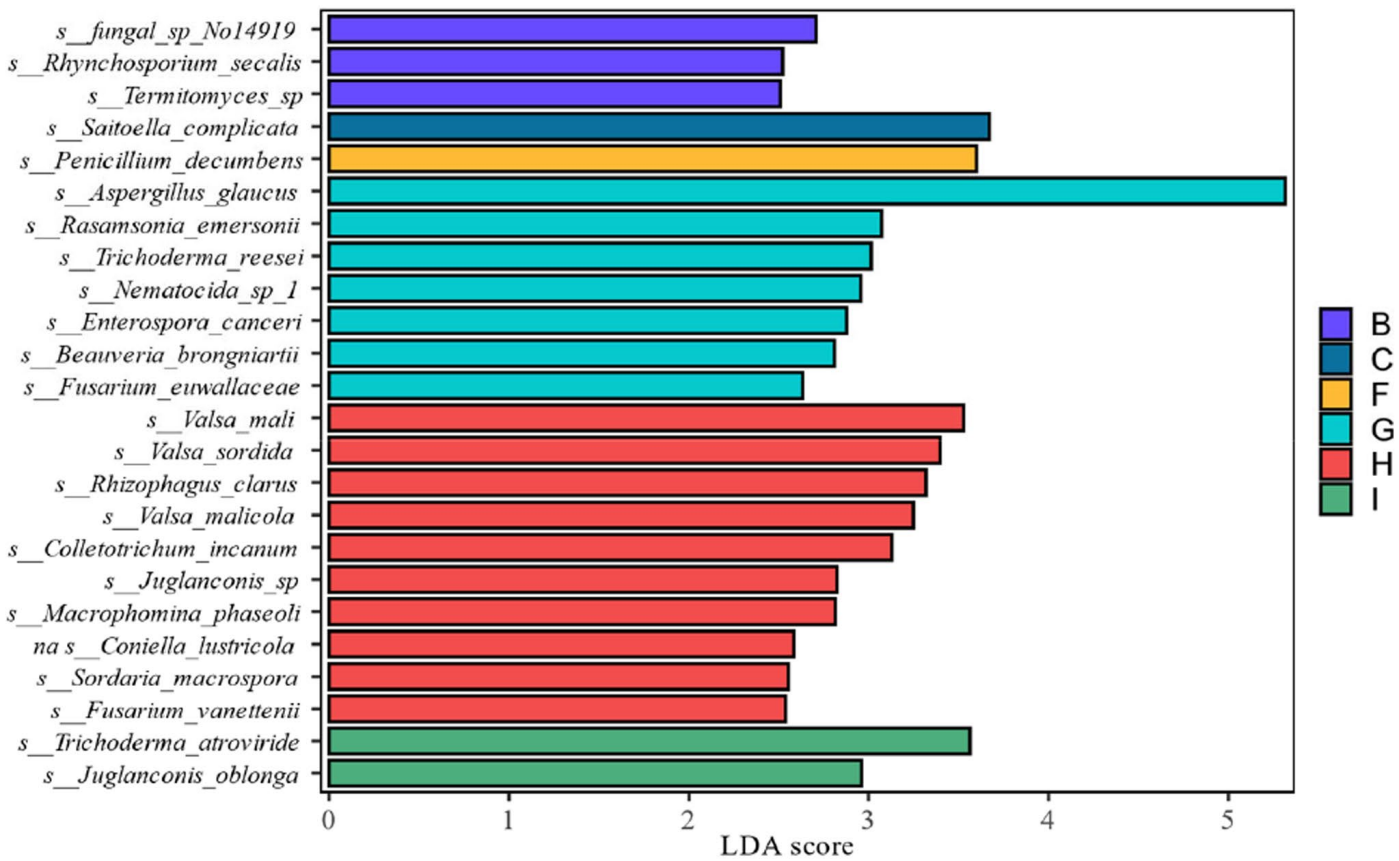
LDA value distribution of different in species different fermentation stages of cigar smoke.

LEfSe analysis as shown in Figure 11 showed that from the species level each fermentation stage fungal flora changed significantly during fermentation. At the species level LDA score ≥ 2.5, *Saitoella_complicata* in FC fermentation stage, *Penicillium_decumbens* in FF fermentation stage, *Aspergillus_glaucus* in FG fermentation stage, and *Trichoderma_atroviride* in FI fermentation stage were far higher than other fermentation stages, and the key fungal communities were mainly concentrated in the late fermentation stage, among which *Aspergillus_glaucus* was the most prominent in the FG fermentation stage. Combining the relative abundance, the degree of variability and the LDA scores, *Penicillium* spp. and *Aspergillus* spp. are the key fungal genera.

### 3.3 Correlation analysis between key flora and main flavor compounds

**Figure 12:**
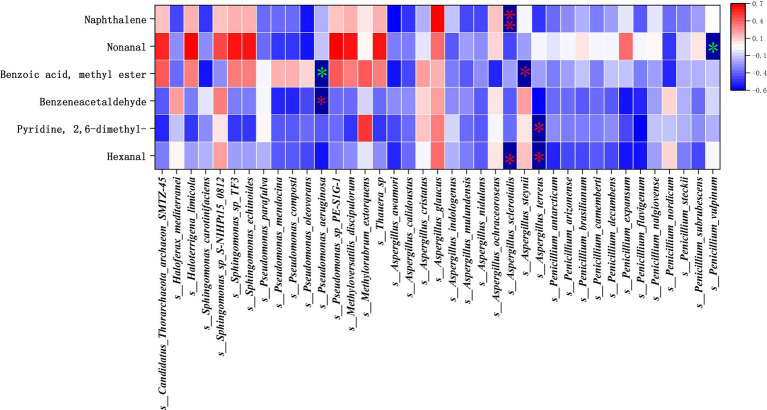
Correlation analysis between key microflora and main flavor substances. *is significant, ** is highly significant, green is positive correlation, red is negative correlation.

Through the studies in the previous two sections, the main microflora and the main flavor substances have been identified, and next, in this section, Pearson correlation analysis was used to further study the correlation between the key bacterial genera and the main aroma substances at different fermentation stages of cigar tobacco leaves. The correlation heat map between key bacterial genera and main body flavor substances at different fermentation stages of cigar tobacco is shown in Figure 12. The effect of archaea on the main body flavor was not significant, the bacterial community *s__Pseudomonas_aeruginosa* showed a significant positive correlation with Benzoic acid, methyl ester (r2 = 0.685), and the fungal community *s__Penicillium_vulpinum* showed a significant positive correlation with Nonanal (r2 = 0.748). The bacterial community *s__Pseudomonas_aeruginosa* showed significant negative correlation with *Benzeneacetaldehyde* (r2 = −0.783), *s__Aspergillus_steynii*, *s__Aspergillus_terreus*, *s__Aspergillus_ sclerotialis* fungi of the genus *Aspergillus* had significant negative correlation (r2 > −0.7) with Hexanal, Pyridine, 2,6-dimethyl-, Benzoic acid, methyl ester, while *s__Aspergillus_ sclerotialis* showed a highly significant negative correlation with Naphthalene (r2 = −0.822). This suggests that *Aspergillus* spp. fungi inhibit the main aroma during fermentation, and *Aspergillus* spp. fungi in the FG fermentation stage affected the accumulation of green, floral, cheesy, and burnt-sweet aromas, while the high activity of the bacterium *Pseudomonas_aeruginosa* in the FG stage was favorable to the accumulation of green aroma, and at the same time inhibited the sweetness. In the FF fermentation stage, the increased activity of the fungus *Penicillium_vulpinum* favors the accumulation of citrus-like aromas.

### 3.4 Analysis of the functional contribution of dominant microorganisms in cigar tobacco leaves

**Figure 13:**
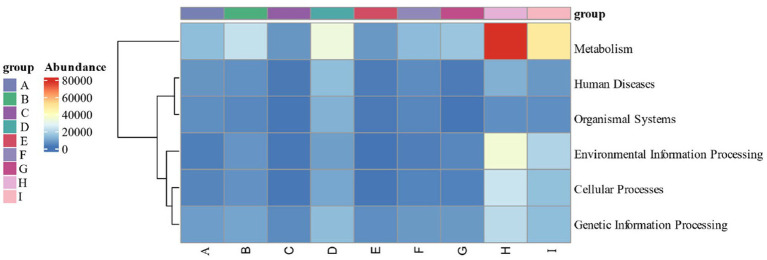
KEGG 7 major metabolic pathways.

**Figure 14:**
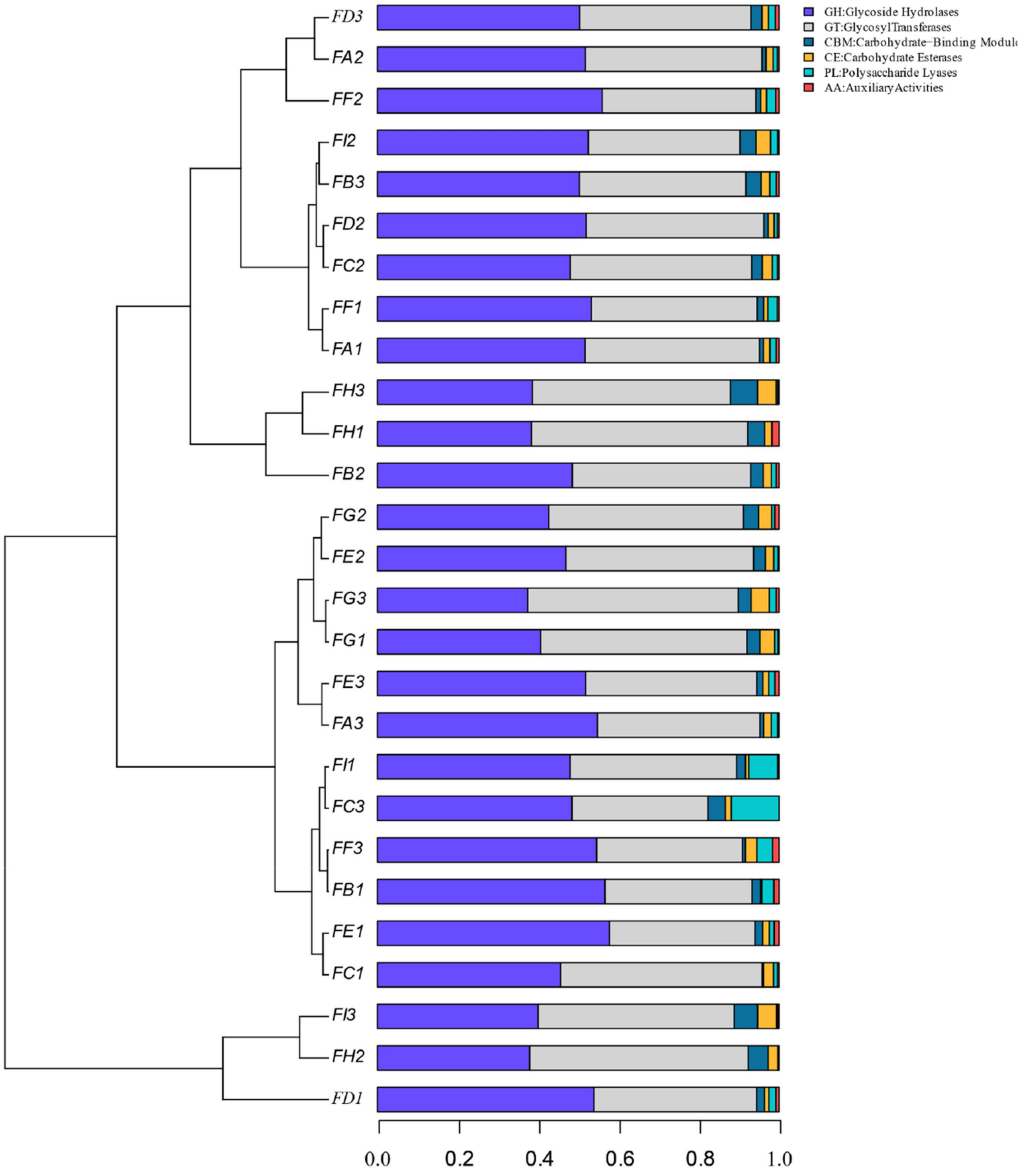
CAZy database of the 6 major carbohydrases.

**Figure 15:**
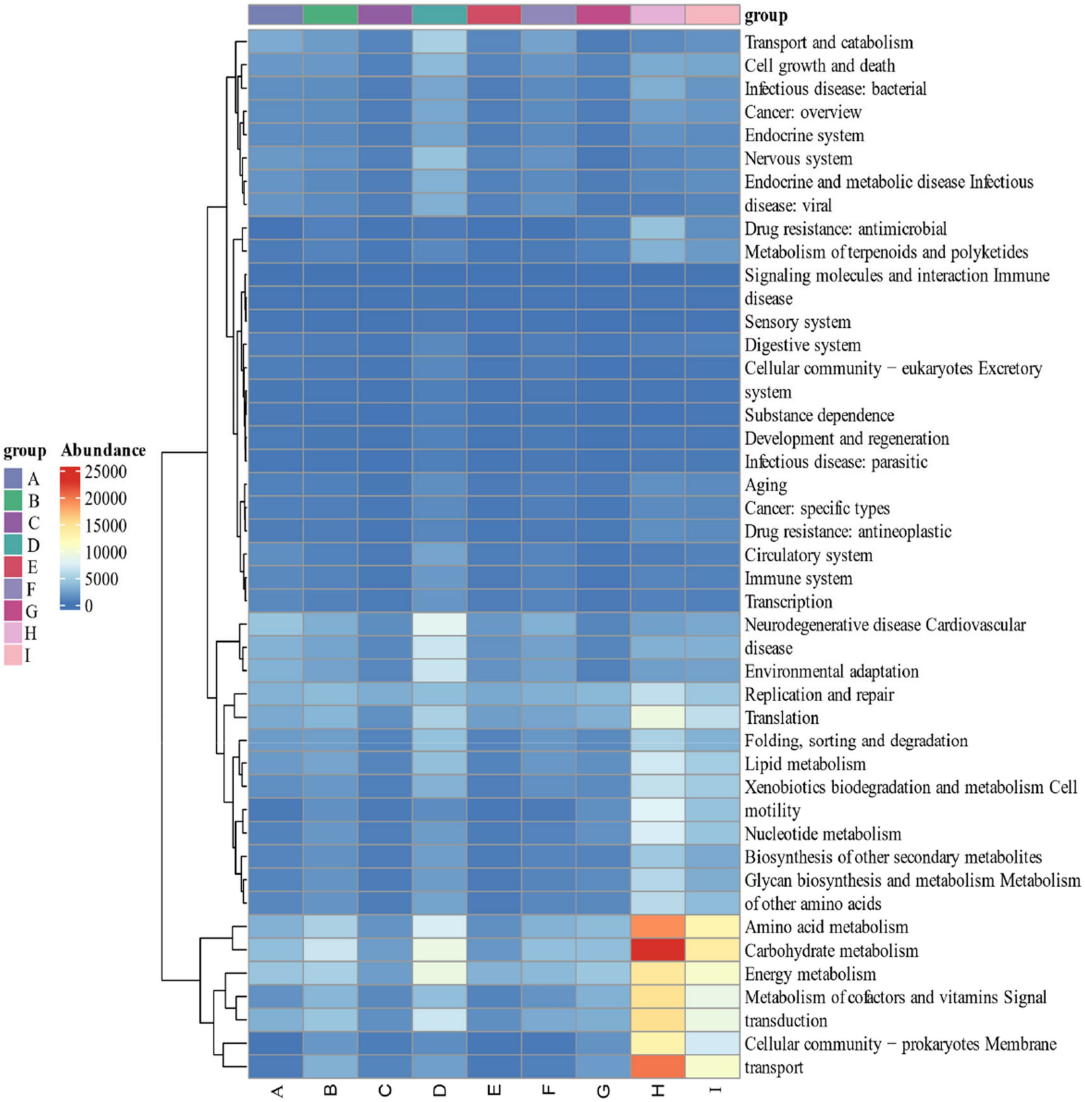
KEGG 43 pathway metabolic pathway.

By calculating the relative abundance of microorganisms in each metabolic pathway at the genus level, it is possible to demonstrate the functional contribution of the cigar tobacco fermentation flora to the KEGG and CAZy metabolic pathways. As shown in Figure 13 KEGG 7 major metabolic pathways, metabolism is the major biometabolic pathway during fermentation. Combined with the annotation results of the 6 major carbohydrate enzymes of the CAZy database in Figure 14, the metabolic enzymes of the genus were mainly dominated by GH: glycoside hydrolase and GT: glycosyltransferase. In addition, the KEGG 43 pathway metabolic pathway was found in Figure 15; amino acid metabolism, carbohydrate metabolism and film transport metabolic pathway were the main metabolic pathways in each fermentation stage of cigar tobacco. The main sources of aroma substances are carotenoid degradation products, Siberian degradation products, aromatic amino acid metabolites, and melanoidin products, which indicates that the main aroma of this paper comes from aromatic amino acid metabolites and melanoidin products, and that the metabolism of bacterial and fungal reproduction during the fermentation of cigar is mainly secreted by glycoside hydrolases and glycosyltransferases to degrade precursor substances.

## 4 Discussion

### 4.1 Major findings

Cigar tobacco is the tobacco with the most distinctive flavor profile, and the fermentation technology and microbial community contribute to the flavor of cigar tobacco leaves (Zheng et al., 2022). In this study, we found that the main flavor profile of cigar tobacco leaves after fermentation was honey-like sweetness, cocoa-flavored burnt-sweetness, citrus-mint-like freshness, and grass-like herbaceousness, and the main flavor substances were hexanal, 2,6-dimethylpyridine, nonanal, phenylglyoxal, naphthalene, and methyl benzoate. During fermentation, the key archaea *Haloferax_mediterranei*, *Haloterrigena_limicola*, *Candidatus_Thorarchaeota_archaeon_SMTZ-45*, and the key bacteria for the *Methyloversatilis* spp., *Sphingomonas* spp., *Thauera* spp., and *s__Pseudomonas* spp., and key fungi were *Penicillium* spp. and *Aspergillus* spp. Correlation analysis of the key colonies with the main flavor substances revealed that the key bacterial and fungal communities were significantly correlated with the main flavor substances, among which the fungi of *Aspergillus* spp. were mainly negatively correlated with Hexanal, Pyridine, 2,6-dimethyl-, Benzoic acid, methyl ester and Naphthalene in the main flavor substances. Naphthalene was negatively correlated with the main aroma and inhibited the accumulation of green, floral, cheesy and caramelized aromas. *s__Pseudomonas_vulpinum* was positively correlated with Nonanal and favored the accumulation of citrus-like aromas. *s__Pseudomonas_aeruginosa* was positively correlated with Benzoic acid, methyl ester, and Naphthalene. aeruginosa was positively correlated with Benzoic acid, methyl ester and negatively correlated with Benzeneacetaldehyde, so that increased activity favored the accumulation of green aroma and inhibited the accumulation of sweet aroma. Functional analysis showed that the dominant bacteria secreted glycoside hydrolases and glycosyltransferases during metabolism, and the metabolic pathways of amino acid metabolism, carbohydrate metabolism and membrane transport were dominant, so the main aroma of this paper was derived from the metabolism of aromatic amino acids, and the products of Meladol.

### 4.2 Analysis of the correlation between microbes and flavor substances in cigar leaves

Currently, there is no systematic study on the mechanism of the influence of its own dominant microorganisms on flavor substances during the fermentation process of cigar tobacco (Evans et al., 2015), and the unique aroma substances are one of the criteria for evaluating the quality of tobacco and distinguishing the quality grade of tobacco, and the main aroma in the present study was the sweet, burnt-sweet, citrus-mint-like fresh and grassy aroma, supplemented by woody, spicy (slightly pungent pepper-like) aroma, floral, cocoa aroma and fruity aroma, which is similar to previous studies. When there is a slight decrease in total nitrogen, nicotine, starch and protein in the chemical composition (Mo, 2017a), it is actually the amino groups on proteins, peptides and amino acids that undergo a non-enzymatic reaction with the carbohydrate carbonyl group (Fu et al., 2019), which enhances the sweet, cocoa-like and milky powder-like aromas (Arsa and Theerakulkait, 2015; Abdelhedi et al., 2017; Lund and Ray, 2017) and also enhances the biological properties and antioxidant activity of milk proteins (Oh et al., 2013; Zhang et al., 2020). Increased clear flavor content is associated with the degradation of chemical components by microbial metabolic enzymes of tobacco leaves. In this study s__Sphingomonas_sp_TF3, *s__Aspergillus_nidulans* and *s__Diaporthe_helianthi* belonging to *Pseudomonas* and *Mycobacterium* spp. which proved to be dominant in roasted and cigarette tobacco studies (Han et al., 2016; Tyx et al., 2016; Chopyk et al., 2017; Smyth et al., 2017). Both can promote decomposition of organic matter (Hang et al., 2018; Li et al., 2019; Zhang et al., 2019; Chen et al., 2020; Kong et al., 2023), and increased levels contribute to the degradation of tobacco chemicals (Liu et al., 2021). Bacteria of the genus *Pseudomonas* (Zhang Y. et al., 2018) in strains with pairs of degradation of methyl benzoate and the production of phenylacetaldehyde ability, and phenylacetaldehyde is formed by the degradation of precursors such as phenylalanine (Zhang Y. et al., 2018). Bacteria of the genus *Sphingomonas* (Liu et al., 2017; Hu et al., 2019) produce aldehydes, esters, and ketones during reproductive metabolism. Fungi of the genus Aspergillus (Victor et al., 2019) produce enzymes that convert fatty acids to Hexanal (hexanal), and lipids are first oxidized by lipoxygenase to form lipid hydroperoxides and then cleaved by hydrogen peroxide lyase to hexanal and other six-carbon aliphatic aromatic compounds. These aldehydes can be isomerized to trans-isomers and then reduced to hexanal (Yin et al., 2022), metabolism produces oxidases, aminotransferases, hydrolases and synthetases to synthesize and decompose Pyridine, 2,6-dimethyl-substances. Enzymes produced by *Penicillium vulpinum* catalyze the oxidative reaction of non-saturated fatty acids into the corresponding aldehydes, palmitoleic acid being the precursor substance of nonanal, and these odorant molecules may interact with nonanal, promoting its formation or altering its pathway of formation. This is consistent with the results of the functional analysis, mainly through two pathways analyzed for aroma production, on the one hand, microorganisms secreted enzymes to degrade amino acids and carbohydrates are two explained small molecules undergo a Merad reaction to produce aroma, and on the other hand, microorganisms directly produce aroma by utilizing and degrading aromatic amino acids, and in this paper, for the first time, we found that microorganisms can produce aroma substances by transporting genes through membranes. It is hypothesized that the process of aroma production may be in the fermentation process, microorganisms will secrete a thin film of biopolymers, covering the surface of cigar tobacco leaves to form a protective layer, microorganisms in the fermentation process will produce genes that represent specific aroma substances, genes are transmitted to the surrounding microorganisms through the film, the microorganisms that receive the genes to synthesize the specific aroma substances, the synthesized aroma substances will be gradually released through the film onto the surface of the cigar leaf. Cigar tobacco leaf surface, the specific mechanism should be further studied to determine.

### 4.3 Future direction

The correlation between key microorganisms and the main flavor substances during the fermentation of cigar tobacco leaves was investigated to further reveal the specific contribution of microorganisms to the formation of odors and to provide a reference for the regulation of odor characteristics during the fermentation process. The popularity of cigar cigarettes among consumers is inseparable from their unique flavor, therefore, it is increasingly important to develop fermentation strains that produce good flavor substances. Based on the present study, we can focus on the screening and development of flavor strains and the synergistic effect between the dominant strains and flavor strains, so as to develop new cigar leaf fermentation agents, which will promote the enhancement of the flavor of cigar and the standardization of the production of cigar tobacco.

## Data availability statement

The original contributions presented in the study are included in the article/supplementary material, further inquiries can be directed to the corresponding author.

## Author contributions

XW: Data curation, Software, Writing - original draft. YH: Conceptualization, Writing - review & editing. QW: Writing - review & editing. JL: Data curation, Software, Writing - review & editing. SF: Writing - review & editing. DH: Conceptualization, Writing - review & editing. XP: Software, Writing - review & editing. JC: Data curation, Writing - review & editing. YG: Data curation, Writing - review & editing. YN: Conceptualization, Writing - review & editing.
